# Immunogenic FEAT protein circulates in the bloodstream of cancer patients

**DOI:** 10.1186/s12967-016-1034-2

**Published:** 2016-09-22

**Authors:** Yan Li, Kyosuke Kobayashi, Marwa M. Mona, Chikako Satomi, Shinji Okano, Hiroyuki Inoue, Kenzaburo Tani, Atsushi Takahashi

**Affiliations:** 1 Division of Molecular and Clinical Genetics, Kyushu University, Fukuoka, Japan; 2 Division of Translational Cancer Research, Medical Institute of Bioregulation, Kyushu University, Fukuoka, Japan; 3 Department of Innovative Applied Oncology, Graduate School of Medical Sciences, Kyushu University, Fukuoka, Japan; 4 Department of Hematology and Oncology, Graduate School of Medicine, Kyoto University, 54 Kawaharacho, Shogoin, Sakyo-ku, Kyoto, 606-8507 Japan; 5 Department of Medical Biochemistry, Faculty of Medicine, Tanta University, Tanta, Egypt; 6 Division of ALA Advanced Medical Research, Institute of Medical Science, University of Tokyo, Tokyo, Japan

**Keywords:** FEAT, METTL13, Immunotherapy, Tumor marker, ELISA, Cancer screening, Cancer prevention

## Abstract

**Background:**

FEAT is an intracellular protein that potently drives tumorigenesis in vivo. It is only weakly expressed in normal human tissues, including the testis. In contrast, FEAT is aberrantly upregulated in most human cancers. The present study was designed to investigate whether FEAT is applicable to tumor immunotherapy and whether FEAT is discernible in the bloodstream as a molecular biomarker of human cancers.

**Methods:**

Two mouse FEAT peptides with predicted affinities for major histocompatibility complex H-2Kb and H-2Db were injected subcutaneously into C57BL/6 mice before subcutaneous transplantation of isogenic B16-F10 melanoma cells. Intracellular localization of FEAT was determined by immunogold electron microscopy. Immunoprecipitation was performed to determine whether FEAT was present in blood from cancer patients. A sandwich enzyme-linked immunosorbent assay was used to measure FEAT concentrations in plasma from 30 cancer patients and eight healthy volunteers.

**Results:**

The vaccination experiments demonstrated that FEAT was immunogenic, and that immune responses against FEAT were induced without deleterious side effects in mice. Electron microscopy revealed localization of FEAT in the cytoplasm, mitochondria, and nucleus. Immunoprecipitation identified FEAT in the blood plasma from cancer patients, while FEAT was not detected in plasma exosomes. Plasma FEAT levels were significantly higher in the presence of cancers.

**Conclusions:**

These findings suggest that FEAT is a candidate for applications in early diagnosis and prevention of some cancers.

## Background

Accumulating evidence has indicated that more than half of cancers are preventable by lifestyle changes and known preventive strategies including screening, vaccines, drugs, and surgical interventions [1]. However, tumorigenesis in humans also involves uncontrollable intrinsic processes, such as heavy mutational burdens owing to random errors in DNA replications and DNA damage caused by endogenous biochemical processes [2]. This view has been supported by the fact that the number of stem cell divisions explains most of the variation in cancer risks among tissues [3]. Although it has been shown that extrinsic factors contribute to 70–90 % of cancer development [4], the “extrinsic” processes include inflammatory mediators, immune responses, hormones, and the tissue microenvironment, which may not be easily modifiable.

Cancers typically develop over 10–30 years, providing a long-term opportunity for intervention. A promising approach for cancer prevention and screening is to target a common molecular marker associated with most tumors [5]. We previously found a tumor-promoting intracellular protein that is upregulated in most human cancers, which is called faint expression in normal tissues, aberrant overexpression in tumors (FEAT). FEAT is encoded by the *METTL13* gene and has S-adenosylmethionine-binding motifs characteristic of methyltransferases. More than 80 % of transgenic mice that express FEAT in the thymus, spleen, liver, and lungs spontaneously develop malignant lymphoma and/or hepatocellular carcinoma. A tissue array study of 168 cancer patients demonstrated upregulation of FEAT in colorectal, pancreatic, prostate, breast, ovary, thyroid, and non-small-cell lung cancers [6]. It has been suggested that miR-16 suppresses tumorigenesis by targeting FEAT [7]. Although a recent report has demonstrated downregulation of FEAT (METTL13) in bladder carcinoma [8], specimens obtained from >3 cm from the tumors were used as normal controls. Multifocal occurrence and frequent recurrence are characteristic features of bladder cancers. A histologically normal but genetically altered area of epithelium has been hypothesized, which later produces multiple, ostensibly independent tumors (“field cancerization”) [9, 10]. Whether FEAT is upregulated in apparently normal tissues as a result of field cancerization is a subject of interest.

Despite widespread overexpression among human cancers, FEAT expression in normal adult tissues is limited to a moderate level in the testis, and weak levels in the brain and liver [6]. Such proteins expressed in cancers and the testis have been pursued as tumor-associated antigens for cancer immunotherapy [11]. Tumor-associated antigens are recognized by the innate and adaptive immune systems and elicit humoral and cellular immune responses that often control or even eliminate cancer cells [12, 13]. A previous SEREX (serological analysis of recombinant cDNA expression libraries) study detected antibodies against FEAT (METTL13) in sera from healthy persons [14], implying a lack of self-tolerance against FEAT. Self proteins to which the immune system is not tolerant are promising targets for immunotherapy [15]. FEAT overexpression in the cytoplasm has also been seen in ductal carcinoma in situ of breast and liver cirrhosis adjacent to hepatocellular carcinoma [6], indicating that the immunotherapy targeting FEAT could potentially also eradicate premalignant lesions.

Although the brain is protected by the blood–brain barrier and the liver has a high regenerative capacity, immune responses may have serious adverse effects when the protein is expressed in a minor subpopulation of cells [16] such as somatic stem cells, which are critical for tissue integrity. This aspect prompted us to test whether induction of cytotoxic T lymphocyte (CTL) reactions against FEAT is deleterious.

Early detection of cervical, colon, lung, and breast cancers reduces disease-specific deaths. However, overdiagnosis of “pseudocancer” is a general concern for sensitive cancer-screening procedures [17, 18]. Nonetheless, detection and characterization is the first step toward the development of biomarkers that stratify patients into risk groups [19]. Conservative approaches such as active surveillance or “watchful waiting” [20] are employed for slow-growing tumors that do not affect a natural lifespan. Endoscopic resection and preventive surgery can be an option for lesions with higher risks. Chemoprevention or preventive therapy, if available [1, 17], may be appropriate for patients with pre-malignant or pre-invasive lesions.

Proteins overexpressed at the early stages of tumorigenesis, released from the cells, and detectable in the serum or plasma are good candidates for molecular biomarkers that assist early diagnosis of cancers [21]. If FEAT is released from cancer cells, blood FEAT may also be a response biomarker for immunotherapies to eradicate FEAT-positive (pre)malignant cells [21]. However, it remains to be clarified whether FEAT is detectable in the blood of cancer patients.

Here we investigated whether FEAT is immunogenic and whether immune responses against intracellular FEAT are deleterious to the host. Our mouse experiments using isogenic tumor transplantation demonstrated that cell-mediated immunity against FEAT can be elicited without serious adverse effects. We further sought to determine whether FEAT is released from cancer cells into the bloodstream. FEAT was detected in the blood plasma from cancer patients and quantifiable by a sandwich enzyme-linked immunosorbent assay (ELISA). Thus, FEAT may facilitate prevention [22] and early detection [21] of some cancers.

## Methods

### Cell culture

HeLa, Lewis lung carcinoma, B16-F1, and B16-F10 cells, obtained from the American Type Culture Collection, were cultured in Dulbecco’s modified Eagle’s medium containing 10 % fetal bovine serum at 37 °C with 5 % CO_2_. Mouse embryonic stem (ES) cells (E14) were cultured in StemMedium Serum Free Media for Mouse ES Cell (DS PHARMA BIOMEDICAL, Suita, Japan) containing 0.1 mM β-mercaptoethanol and ESGRO Leukemia Inhibitory Factor (Chemicon, Merck Millipore, Darmstadt, Germany) on tissue culture dishes gelatinized using ESGRO Complete Gelatin Solution (Merck Millipore).

### Generation of a rabbit anti-mouse FEAT antibody

A rabbit polyclonal antibody was produced and affinity purified by Eurofins Genomics (Tokyo, Japan) using His-tagged mouse FEAT purified with TALON Metal Affinity Resin (Clontech Laboratories, Takara Bio, Otsu, Japan) under denaturing conditions.

### Selection and synthesis of major histocompatibility complex (MHC) class I-restricted peptides

Prediction of 9-mer and 10-mer peptides with high affinity for H-2Kb and H-2Db was performed using the mouse FEAT (Mettl13) sequence and BIMAS [23] and SYFPEITHI [24] software. Peptides with a purity of >90 % were synthesized by Eurofins Genomics.

### Mouse experiments

Female C57BL/6J mice (6 weeks old) were purchased from Charles River Japan (Yokohama, Japan). Experiments were approved by an animal experiment committee at Kyushu University and performed in accordance with national and institutional guidelines for animal use in research.

Immunization was conducted subcutaneously with 20 μg peptide A (EWYGTYLEL) and/or 20 μg peptide B (ALLRNPELL) in 100 μl phosphate-buffered saline (PBS) containing 10 μg AbISCO-100 adjuvant (Isconova, Novavax, Gaithersburg, MD, USA) in the left flank twice at a 1-week interval. Control mice only received PBS or the adjuvant. One week after the second immunization the mice were injected subcutaneously with 1 × 10^5^ B16-F10 cells into their right flank. The tumor size was monitored daily. Mice were sacrificed when the largest tumor in the experiment reached 10 mm in diameter. Vaccination experiments were conducted four times using three mice per treatment group.

Mice were euthanized by neck dislocation, dissected, and fixed with 3.7 % formaldehyde in PBS. Tissues were cut into 2-mm-thick sections and placed into tissue cassettes (Tissue-Tek Uni-Cassette, Sakura Finetek Japan). Fixed tissues were embedded in paraffin, sectioned with a microtome, and stained with hematoxylin and eosin (H&E) by the Laboratory of Technology, Medical Institute of Bioregulation, Kyushu University.

Blood samples were collected from the peri-orbital sinus of mice, allowed to clot, and centrifuged at 800×*g* for 15 min. Serum aspartate aminotransferase (AST)/glutamate oxaloacetate transaminase (GOT), alanine aminotransferase (ALT)/glutamate pyruvate transaminase (GPT), and creatinine were measured with a FUJI DRI-CHEM 3500v and FUJI DRI-CHEM slides (Fujifilm, Tokyo, Japan).

### Immunohistochemistry

Paraffin-embedded sections were deparaffinized with Clear-Advantage (Polysciences, Warrington, PA, USA), rehydrated, treated with Citrate-based Antigen Unmasking Solution (Vector laboratories, Burlingame, CA, USA), and stained with a mouse anti-CD3-ζ monoclonal antibody (6B10.2) (1:500 dilution; Santa Cruz Biotechnology, Dallas, TX, USA) and a rabbit polyclonal anti-CD8 antibody (1:200 dilution; Bioss Antibodies, Woburn, MA, USA) using a Multiview (mouse-HRP/rabbit-AP) IHC kit and IHC background blocker (Enzo Life Sciences, Farmingdale, NY, USA). The sections were counterstained with Mayer’s Hematoxylin (Merck), and coverslips were mounted with CC/Mount Aqueous Permanent Mounting Medium (Diagnostic BioSystems, Pleasanton, CA, USA). Images were acquired using a BZ-9000 Fluorescence Microscope (KEYENCE, Osaka, Japan) and a Zeiss Axioskop 2 plus microscope equipped with a Zeiss AxioCam camera controlled by AxioVision software (Carl Zeiss Microscopy, Jena, Germany).

### Heparinized human blood plasma from healthy volunteers

After receiving written informed consent, blood was collected from three healthy volunteers by venipuncture into a tube containing sodium heparin and centrifuged at 1200 rpm for 10 min at 21 °C. Five samples of normal human plasma in sodium heparin were purchased from Cosmo Bio (Tokyo, Japan).

### Immunoprecipitation

ImmunoCruz IP/WB reagents (Santa Cruz Biotechnology) were used to immunoprecipitate FEAT from 1 mg of plasma.

### Immunoblotting

Proteins were subjected to sodium dodecyl sulfate-polyacrylamide gel electrophoresis (SDS-PAGE) and semidry transfer as described previously [6]. The membrane was stained with primary and secondary antibodies using Can Get Signal Immunoreaction Enhancer Solution (TOYOBO, Osaka, Japan). Signals were visualized with Amersham ECL Select Western blotting detection reagent and an ImageQuant LAS 500 (GE Healthcare Life Sciences, Little Chalfont, UK). Antibodies used were: a rabbit polyclonal antibody against peroxiredoxin 1 (Atlas Antibodies, Stockholm, Sweden) and mouse monoclonal antibodies against β-actin (Santa Cruz Biotechnology) and FEATΔC (7F2) (MBL, Nagoya, Japan).

### Immunogold electron microscopy

Adherent cells were washed once with PBS and exposed to a prefixation solution (4 % paraformaldehyde, 0.4 % glutaraldehyde, 3.4 % sucrose, and 3 mM CaCl_2_ in 0.1 M cacodylate buffer, pH 7.4) for 10 min at room temperature. The cells were detached by a cell scraper (Nunc, Thermo Scientific) and centrifuged at 2000 rpm for 10 min in a swing-bucket rotor. After overnight fixation at 4 °C, the cell pellet was washed with PBS for 1 h at room temperature, dehydrated in a graded series of ethanol solutions on ice, embedded in LR White resin (Medium grade, Electron Microscopy Sciences, Hatfield, PA, USA) at −20 °C, exposed to ultraviolet radiation for 3 days at −20 °C, and then incubated for 24 h at 45 °C. Resin-embedded cells were sectioned at 100-nm thicknesses using a Leica EM UC7 Ultramicrotome (Leica Microsystems, Wetzlar, Germany) and collected on Formvar carbon-coated nickel grids. Grids were blocked with 3 % bovine serum albumin (BSA)-PBS for 15 min at room temperature, stained with rabbit anti-human FEATΔN or anti-mouse FEAT antibodies in 0.3 % BSA-PBS overnight at 4 °C, and then incubated with colloidal gold (10-nm)-conjugated goat anti-rabbit IgG (1:50 dilution in PBS; EY Laboratories, San Mateo, CA, USA) for 2 h at room temperature. Postfixation was performed with 0.5 % osmium tetroxide in PBS for 5 min at room temperature, followed by staining with 2 % uranyl acetate for 5 min to confer a light contrast. Cell sections were examined using a Tecnai 20 transmission electron microscope (FEI, Hillsboro, OR, USA). Images were acquired with an Eagle 2k CCD camera with a high resolution scintillator (FEI).

### ELISA

A solid phase sandwich ELISA kit for human FEAT (FEAT Assay Kit; Lot. 1H-512 and 1L-517) was produced by the contract manufacturing service of Immuno-Biological Laboratories (Fujioka, Japan). To 96-well plates coated with the rabbit polyclonal anti-human FEATΔC antibody, 100 μl EIA buffer (1 % BSA and 0.05 % Tween-20 in PBS) and 100 μl sample were added, followed by overnight incubation at 4 °C. The plates were treated with 100 μl/well mouse anti-human FEATΔC monoclonal antibody (MBL) for 30 min at 37 °C, 100 μl/well anti-mouse IgG (H + L) goat IgG Fab′ conjugated with horseradish peroxidase for 30 min at 37 °C, and then 100 μl/well tetramethylbenzidine solution for 30 min at room temperature. The reaction was stopped with 100 μl/well 1 N H_2_SO_4_. Optical density was read at 450 nm using an EnSpire Multimode Plate Reader (PerkinElmer, Waltham, MA, USA). Purified His-tagged recombinant human FEAT was used to generate a standard curve.

Plasma C-reactive protein (CRP) was measured with a Quantikine ELISA Human CRP Immunoassay kit (R&D Systems, Minneapolis, MN, USA).

### Exosome purification

Exosomes were purified from plasma using a Total Exosome Isolation (from plasma) kit (Invitrogen, Thermo Fisher Scientific, Waltham, MA, USA). Exosomes in the pellet were resuspended in SDS-PAGE sample buffer and incubated for 3 min at 95 °C.

### Statistical analysis

Statistical analyses were performed using the Statcel4 add-in package (OMS Publishing, Tokorozawa, Japan) for Microsoft Excel. The Kruskal–Wallis test, a non-parametric one-way analysis of variance, was used for AST/GOT and ALT/GPT because homogeneity of variances was unlikely according to Bartlett’s test. The Kruskal–Wallis test followed by the Steel test, a multiple comparison test for non-parametric data, were used for ELISA data, because a normal distribution was unlikely according to Pearson’s Chi-square goodness-of-fit test.

## Results

### Immune responses induced in mice by FEAT peptides

We developed a novel antibody that recognized mouse FEAT, because the available antibodies against human FEAT are not cross-reactive with mouse FEAT. The antibody detected mouse FEAT in Lewis lung carcinoma, B16-F1 melanoma, and B16-F10 melanoma cells (data not shown), indicating the feasibility of these C57BL/6 mouse-derived cell lines as cellular targets for CTL responses against FEAT.

CTL-epitope peptides from tumor-associated antigens have been used to treat melanoma patients [25]. BIMAS [23] and SYFPEITHI [24] software, which have been widely validated in vitro [26], selected two peptides, named A and B, which were predicted to have affinities for H-2Kb and H-2Db, respectively, for presentation to CTLs by dendritic cells [26].

**Fig. 1:**
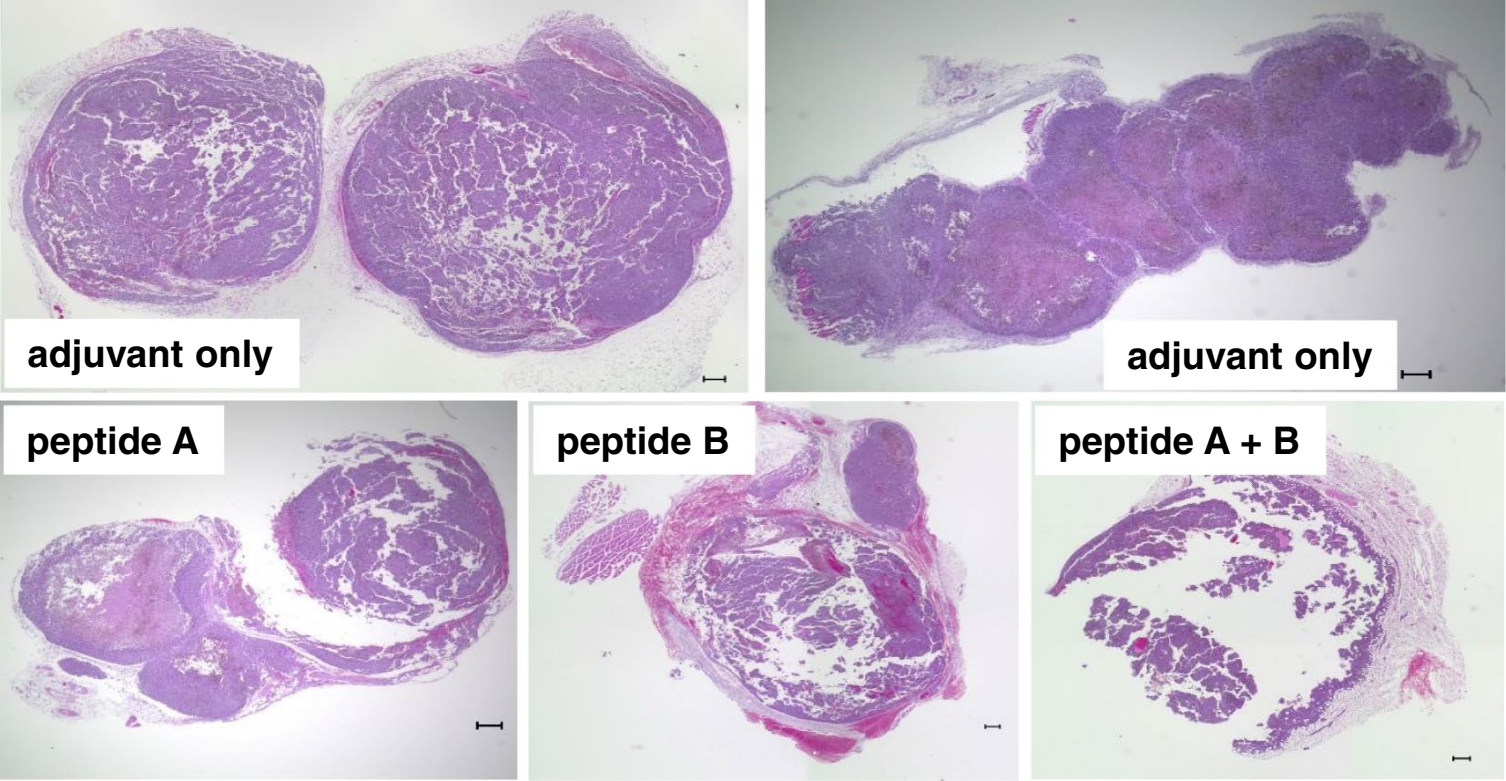
Representative images of B16-F10 tumors that developed in mice. Tumors formed by transplanted B6-F10 cells were stained with H&E. Note the necrosis and artifactual destruction of friable tumors despite sectioning after fixation. *Scale bars* 300 μm

**Fig. 2:**
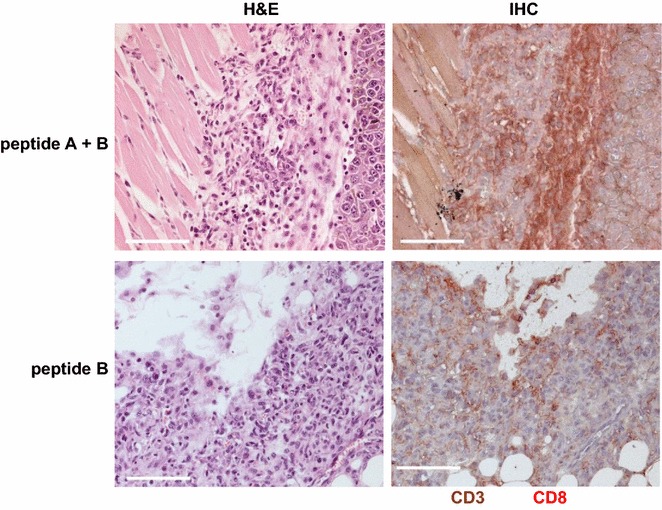
Effects of FEAT peptides on B16-F10 tumors. *Left panels* Representative images of H&E-stained sections of tumors. *Right panels* Double immunohistochemical staining for CD3 (*brown*) and CD8 (*red*). *Scale bars* 100 μm

Mice were immunized with peptides A and/or B in combination with AbISCO-100 adjuvant based on Quillaja saponin mixed with cholesterols and phospholipids. Next, the animals were challenged subcutaneously with B16-F10 cells and sacrificed at 9–17 days after transplantation. Peptides or adjuvant did not affect the growth of tumors as estimated by tumor sizes. Interestingly, however, tumors were soft and friable in mice injected with peptide A and/or B (Fig. 1) in contrast to rubbery hard tumors in mice injected with the adjuvant alone. These results suggested that at least some tumor cells had died because of immune responses to the peptides and some cells that underwent immunoediting had survived and proliferated. H&E-stained sections revealed that the peptides induced infiltration of lymphocytes, neutrophils, and macrophages. In particular, marked infiltration of lymphocytes at the periphery of tumors was observed in mice injected with both peptide A and B (Fig. 2, upper left panel), as supported by immunohistochemical staining for CD3 and CD8 (Fig. 2, upper right panel). However, infiltration of macrophages and proliferation of fibroblasts were predominant in some tumors (Fig. 2, lower panels), illustrating immunosuppressive environments mediated by myeloid-derived suppressor cells [27] and tumor-associated macrophages [28].

**Fig. 3:**
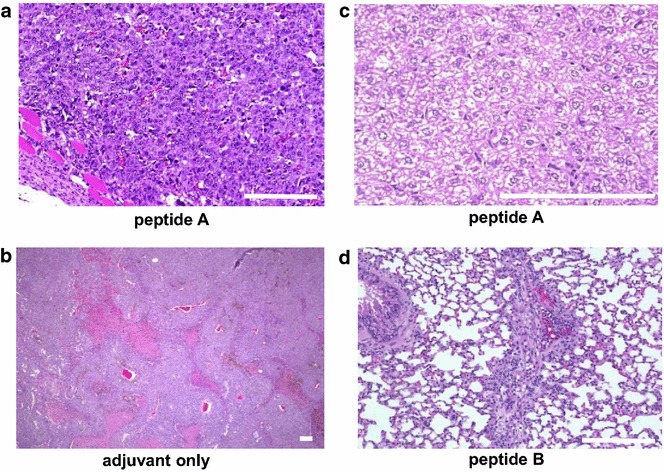
Effects of FEAT peptides on B16-F10 tumors and normal tissues. H&E staining of tumors (**a**, **b**), liver (**c**), and lungs (**d**). *Scale bars* 100 μm

Tumors in mice injected with the peptides had fewer and smaller blood vessels (Fig. 3a) than controls (Fig. 3b), suggesting anti-angiogenic effects of the immunization, and activation of Kupffer cells in the liver (Fig. 3c). These results indicate that the peptides caused systemic immune responses against the FEAT peptides.

### Adverse effects of immune responses against FEAT

**Fig. 4:**
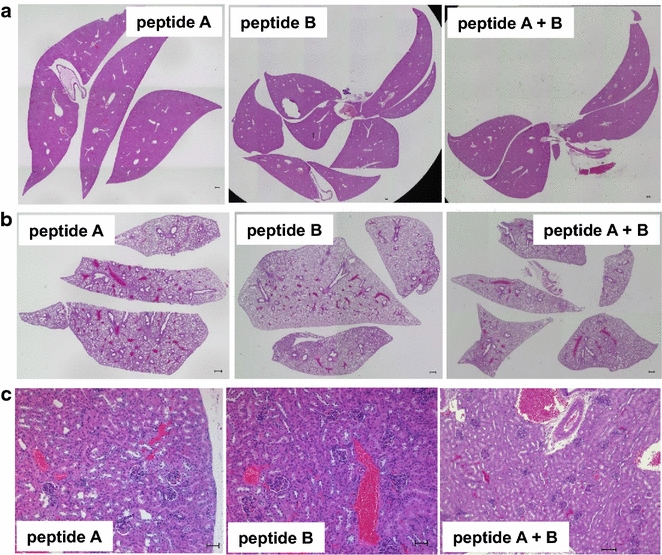
Representative sections of the liver, lungs, and kidneys from mice. The liver (**a**), lungs (**b**), and kidneys (**c**) of the same mouse shown in Fig. 1 were stained with H&E. *Scale bars* (**a**, **b**) 300 μm and (**c**) 100 μm

**Fig. 5:**
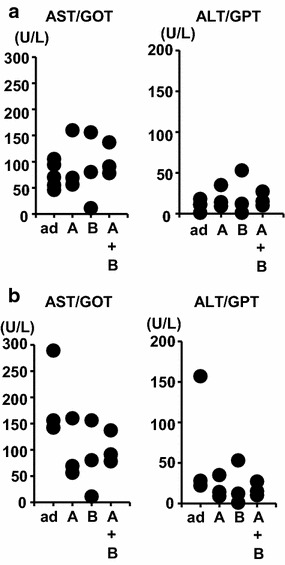
Plasma AST/GOT and ALT/GPT of mice injected with peptides. Each *dot* represents a sample at 7 days (**a**) and 23 days (**b**) after the first injection of adjuvant with or without peptides (*P* > 0.3, Kruskal–Wallis test). *ad* adjuvant only, *A* peptide A, *B* peptide B, *A* *+* *B* peptide A and peptide B

The peptide vaccine was well tolerated without serious adverse effects as examined by overall behavior, body weight, macroscopic observations at dissection, H&E staining of the liver (Fig. 4a) and lungs (Fig. 4b), and analyses of serum AST/GOT and ALT/GPT at 7 and 23 days after peptide injection (Fig. 5). The kidneys in all mice showed no abnormal histological changes (Fig. 4c), and mice had normal serum creatinine levels of 0.1–0.3 mg/dl. We did not notice any correlation of AST/GOT and ALT/GPT with tumor sizes. Periarteriolar lymphocyte infiltration was noted in the lungs of a mouse injected with peptide B (Fig. 3d), indicative of an autoimmune response. However, no signs of respiratory distress were noted. These results suggest that immune responses against FEAT are not deleterious to normal tissues, and that immunotherapy targeting FEAT is possible without serious adverse events in the host.

### Intracellular localization of FEAT protein

**Fig. 6:**
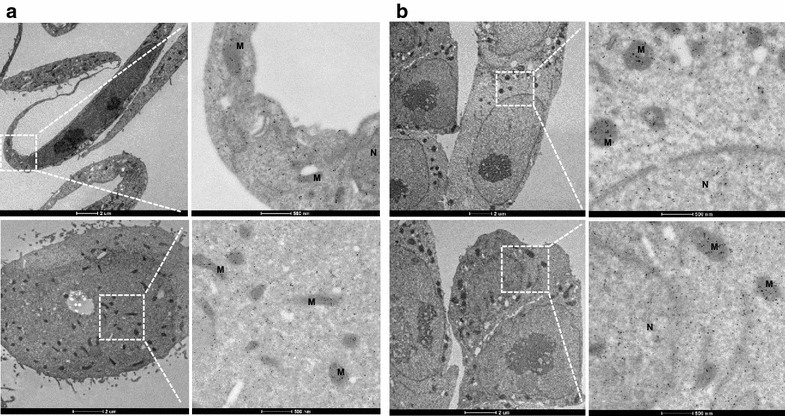
Transmission electron microscopy of FEAT intracellular localization. Immunogold labeling of FEAT in HeLa (**a**) and mouse ES cells (**b**). *Scale bars* 2 μm (*left panels*) and 500 nm (*right panels*). *M* mitochondria, *N* nucleus

Previous immunofluorescence studies have shown diffuse localization of FEAT in the cytoplasm and nucleus [6]. To assess whether FEAT is present in secretory vesicles, we performed immunogold electron microscopy. FEAT was detected in the cytoplasm, mitochondria, and nucleus of HeLa cells (Fig. 6a) and mouse ES cells (Fig. 6b). The specificity of the immunogold staining with the rabbit anti-mouse FEAT antibody was evaluated by parallel staining of FEAT-deficient (*Mettl13*^−/−^) ES cells (KK and AT, unpublished observations). The results were contradictory to the possibility of FEAT secretion by the conventional secretory pathway.

### Detection of FEAT in the blood plasma of cancer patients

**Fig. 7:**
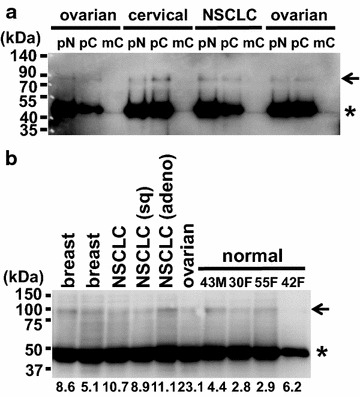
Immunoprecipitation of FEAT from blood plasma. FEAT was immunoprecipitated from the plasma of healthy volunteers and patients with the indicated cancers using rabbit polyclonal anti-human FEATΔN (**a**, pN; **b**) and anti-human FEATΔC (**a**, pC) antibodies and a mouse anti-human FEATΔC monoclonal antibody (**a**, mC). Blots are probed with the rabbit polyclonal anti-human FEATΔN antibody. *Arrows* indicate FEAT protein. *Asterisks* denote heavy chains of IgG. **b** Plasma FEAT concentrations (ng/ml) measured by ELISA are shown *below* the *panels*. The age and sex of healthy volunteers are indicated. *NSCLC* non-small cell lung cancer, *sq* squamous cell carcinoma, *adeno* adenocarcinoma

Previously, we found that caspase-3 cleaves human FEAT after Asp112, Asp274, and Asp288. We raised antibodies against two major fragments produced by caspase-3, FEATΔN (amino acids 289–699 of human FEAT) and FEATΔC (amino acids 1–274) [6]. First, we tested whether these antibodies immunoprecipitated FEAT, if present, from the plasma of cancer patients. FEAT was immunoprecipitated from plasma with rabbit anti-human FEATΔN and FEATΔC antibodies, but not with the mouse anti-human FEATΔC monoclonal antibody (Fig. 7a). This result indicates that blood contains FEAT detectable by specific antibodies.

**Fig. 8:**
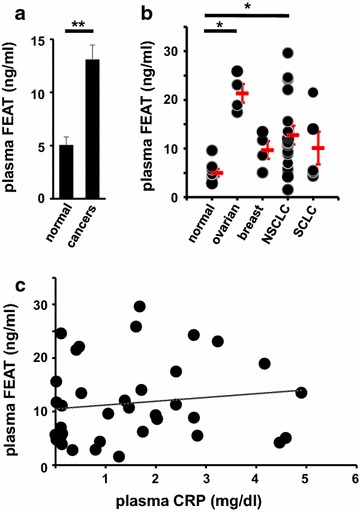
Plasma FEAT concentrations in healthy volunteers and cancer patients. **a** Values denote the mean ± SEM of plasma FEAT in healthy volunteers (*n* = 8) and cancer patients (*n* = 30). **P < 0.01, Welch’s t test. **b** *Each dot* represents a sample, while the *red bars* indicate the mean ± SEM (*P* < 0.01, Kruskal–Wallis test). *NSCLC* non-small cell lung cancer, *SCLC* small cell lung cancer. *P < 0.05, Steel’s test. **c** Correlation between plasma CRP and plasma FEAT. A regression line is shown (Spearman correlation coefficient = 0.18; *P* > 0.26)

To quantify FEAT in the plasma samples, we developed a sandwich ELISA in which FEAT was captured by the rabbit anti-human FEATΔC antibody. The mouse anti-human FEATΔC monoclonal antibody, which was highly specific as shown by immunoblotting (data not shown), was used for detection. We chose non-gastrointestinal cancers because non-radiological screening procedures are available for common gastrointestinal cancers (i.e., colorectal, gastric, esophageal, hepatic, and pancreatic cancers) such as fecal occult blood tests, endoscopy, and ultrasonography. Plasma was analyzed from 30 cancer patients and eight healthy volunteers (Table 1). All patients had advanced cancer with distant metastasis. Plasma FEAT concentrations were significantly higher in cancer patients than healthy individuals (Fig. 8a), particularly those with ovarian and non-small cell lung cancers (Fig. 8b). The presence of FEAT in some samples was confirmed by immunoprecipitation (Fig. 7b). We did not notice any correlation of FEAT concentrations with tumor burden or cancer aggressiveness. These findings suggest that FEAT is released from cancers, and that plasma FEAT is exploitable as a risk biomarker [21] for FEAT-expressing tumors.

**Table 1:** Characteristics of cancer patients and healthy volunteers

Tumor type	Age, median (range)	Male	Female
Breast	56 (36–57)		4
Ovarian	54 (50–57)		4
Lung			
Non-small cell	59 (37–80)	10	7
Small cell	62 (57–69)	5	
None (normal volunteer)	36.5 (24–55)	3	5

To test the possibility that intracellular FEAT is released by inflammation, we measured CRP in the plasma samples. FEAT levels were not significantly correlated with plasma CRP (Fig. 8c).

**Fig. 9:**
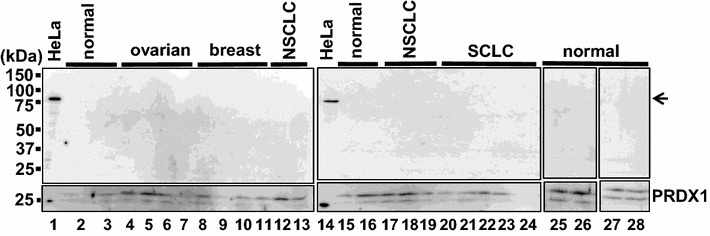
Immunoblotting of plasma exosomes for FEAT. Exosomes isolated from eight healthy volunteers and 18 cancer patients were analyzed by immunoblotting using rabbit anti-human FEATΔC (*upper panel*) and anti-peroxiredoxin 1 (PRDX1) (*lower panel*) antibodies. *HeLa* lysates from HeLa cells. The *arrow* denotes FEAT

Released cytoplasmic proteins may be enclosed in extracellular vesicles called exosomes that are produced by the fusion of multivesicular endosomes with the plasma membrane [29]. Exosomal cancer biomarkers are a topic of intensive research because circulating exosomes are potential sources for liquid biopsies [30]. A previous report identified FEAT (CGI-01; KIAA0859; METTL13) in human thymic exosomes [31]. Exosomes may lyse in the extracellular space via an unknown mechanism. To assess whether FEAT enclosed in exosomes circulates in the bloodstream, we isolated exosomes from the plasma of 18 cancer patients and eight healthy volunteers. Purification was confirmed by immunoblotting for peroxiredoxin 1 [32]. However, FEAT was not detected in the plasma exosomes (Fig. 9), excluding the utility of exosomal FEAT as a cancer biomarker. These results support the notion that FEAT is released by disruption of the plasma membrane [33].

## Discussion

In an effort to translate the cancer biology of FEAT into therapeutic and diagnostic applications, we demonstrated that induction of immune responses against FEAT did not cause serious adverse effects in mice. We also found that FEAT circulates in the bloodstream of cancer patients. FEAT was not detected in plasma exosomes, and blood FEAT was amenable to quantification by sandwich ELISA using anti-FEAT antibodies.

With the advent of sensitive screening procedures, the anxiety of cancer diagnosis may become a psychosocial problem for some cancers that are not detectable by diagnostic imaging and endoscopy (“detected cancer that cannot be found”) [21]. Thus, it is desirable to develop secondary prevention of cancer in parallel. Chemoprevention and “cancer-preventive vaccines” [22, 34] can treat such patients. Although neoantigens due to random mutations have been shown to elicit stronger immune responses than shared antigens [16], including FEAT, such neoepitopes are highly patient-specific and difficult to use for preventive vaccines.

FEAT was shown to be immunogenic by histological analyses. It is notable that the combination of two MHC class I-restricted FEAT peptides stimulated the infiltration of lymphocytes surrounding B16-F10 melanoma cells. Indeed, the presence of tumor-infiltrating lymphocytes is a prerequisite for responses to immune checkpoint inhibitors such as antibodies against CTLA-4, PD-1, and PD-L1 [35]. Combined use of peptides and an immune checkpoint inhibitor [36] may address the limited immune responses in the present study, which were insufficient to eradicate the tumors [37]. The NetMHCpan 2.8 program [38], which has been the best validated in vitro [26], predicted that the peptides A and B are weak and high binders, respectively. An additional three and two high binders for H-2Kb and H-2Db, respectively, were selected from mouse FEAT by the program, raising the possibility that stronger CTL responses can be triggered by the combination of additional peptides. Further studies are necessary to determine whether such robust T cell responses lead to severe toxicity.

The limitations of short peptide vaccines restricting their effectiveness include poor migration of dendritic cells to regional lymph nodes, tolerance induction by nonprofessional antigen-presenting cells, and the lack of CD4+ T cell responses that support CTL activation and maintenance of CD8+ memory T cells [39]. Synthetic long peptides have been exploited to circumvent tolerance induction and activate CD4+ helper T cells [39]. Tumors create immunosuppressive environments that recruit regulatory T cells [40], myeloid-derived suppressor cells [27], and tumor-associated macrophages [28]. Indeed, infiltration of neutrophils and macrophages was observed around B16-F10 tumors. Use of a water-in-oil emulsion such as incomplete Freund’s adjuvant has been associated with recruitment of CTLs to sites of immunization rather than the tumor [41]. However, the present study used the AbISCO-100 adjuvant that does not require emulsification of antigens. It remains to be clarified whether the new generation of immune-stimulating complex adjuvants have a similar problem (i.e., recruitment and apoptosis of CTLs).

Significantly higher plasma FEAT levels in cancer patients than healthy subjects suggest that FEAT is released from the cytoplasm of tumor cells. Tumors without intensive angiogenesis may undergo hypoxia-induced necrosis that releases intracytoplasmic proteins [33]. In addition, cancers are often associated with chronic inflammation, leading to inflammation-associated programmed necrosis such as necroptosis [42] and pyroptosis [43]. However, we could not find a correlation between plasma CRP and plasma FEAT, implying that the release of cellular FEAT does not require intense inflammatory reactions. Further studies should provide insights into the unconventional protein secretion [44] of cytoplasmic FEAT.

FEAT circulating in the blood of cancer patients could facilitate the diagnosis of some cancers, which would be dependent on whether it becomes detectable in the early stages of tumorigenesis. Detection of plasma or serum FEAT by sandwich ELISA is noninvasive, convenient, and inexpensive, supporting the feasibility for repeated screening of the general population [21]. Screening is more efficient and economical with increased incidence of the target disease in the population. If FEAT is released from a wide range of cancers, screening would provide an advantage over other markers applicable to one or a few types of cancer, such as prostate-specific antigen [17]. However, it should be noted that cancers are highly heterogeneous [45], and the secretion of cytoplasmic FEAT may be limited to certain cancer subtypes. In addition to further studies involving hundreds of healthy volunteers and patients with various stages of cancers, analyses of patients with non-cancerous disorders are required to demonstrate the diagnostic efficacy of circulating FEAT. Nonetheless, even if blood FEAT increases in non-cancerous diseases, serial measurements of blood FEAT might be useful for the follow-up of high-risk individuals such as patients with BRCA1/2 mutations for development of breast and ovarian cancers [46].

A possible limitation of blood FEAT is the inability to predict localization of cancers. However, high levels of FEAT in the bloodstream may warrant intensive whole body diagnostic imaging if the elevation of blood FEAT is highly specific for cancer. Another limitation of the current ELISA technique is reduced resolution at <10 ng/ml. This limitation hindered our efforts to determine a cutoff level that discriminated between cancer patients and healthy volunteers. With integrated efforts to increase the sensitivity, the FEAT sandwich ELISA could serve as a promising test for cancer screening.

## Conclusions

In summary, our encouraging preliminary data suggest that immunogenic FEAT protein circulating in the bloodstream provides a resource for applications in early diagnosis and secondary prevention of some cancers [47].

## Abbreviations

AST: aspartate aminotransferase
ALT: alanine aminotransferase
BSA: bovine serum albumin
CRP: C-reactive protein
CTL: cytotoxic T lymphocyte
ELISA: enzyme-linked immunosorbent assay
ES cell: embryonic stem cell
GOT: glutamate oxaloacetate transaminase
GPT: glutamate pyruvate transaminase
H&E: hematoxylin and eosin
MHC: major histocompatibility complex
PBS: phosphate-buffered saline
SDS-PAGE: sodium dodecyl sulfate-polyacrylamide gel electrophoresis
